# Poly[μ_2_-aqua-μ_4_-(2-{3-[(6-chloro­pyridin-3-yl)meth­yl]-2-oxoimidazolidin-1-yl}acetato)-sodium]

**DOI:** 10.1107/S1600536812025007

**Published:** 2012-06-13

**Authors:** Rajni Kant, Vivek K. Gupta, Kamini Kapoor, Chetan S. Shripanavar, Kaushik Banerjee, Madhukar B. Deshmukh

**Affiliations:** a X-ray Crystallography Laboratory, Post-Graduate Department of Physics & Electronics, University of Jammu, Jammu Tawi 180 006, India; b Department of Chemistry, Shivaji University, Kolhapur, 416 004, India; c National Research Centre for Grapes, Pune 412 307, India

## Abstract

In the title compound, [Na(C_11_H_11_ClN_3_O_3_)(H_2_O)]_*n*_, there are two independent Na^I^ ions, one of which lies on an inversion center and is coordinated in a slightly distorted octa­hedral environment. The other Na^I^ ion lies on a twofold rotation axis and is cooordinated in a slightly distorted trigonal–bipyramidal coordination environment. In the organic ligand, the imidazolidine ring adopts a half-chair conformation. The Na^I^ ions bridge organic ligands and water mol­ecules, forming a two-dimensional structure parallel to (100). There are inter­molecular O—H⋯O and weak C—H⋯O hydrogen bonds within the two-dimensional structure.

## Related literature

For background to the insecticidal applications of imidacloprid {systematic name: *N*-[1-[(6-chloro-3-pyrid­yl)meth­yl]-4,5-dihydro­imidazol-2-yl]nitramide}, see: Legocki & Polec (2008 ▶); Kovganko & Kashkan (2004 ▶); Zhao *et al.* (2009 ▶); Tanner *et al.* (2010 ▶); Xu *et al.* (2010 ▶). For ring conformations, see: Duax & Norton (1975 ▶). For related structures, see: Kapoor *et al.* (2011 ▶, 2012 ▶); Kant *et al.* (2012 ▶). 
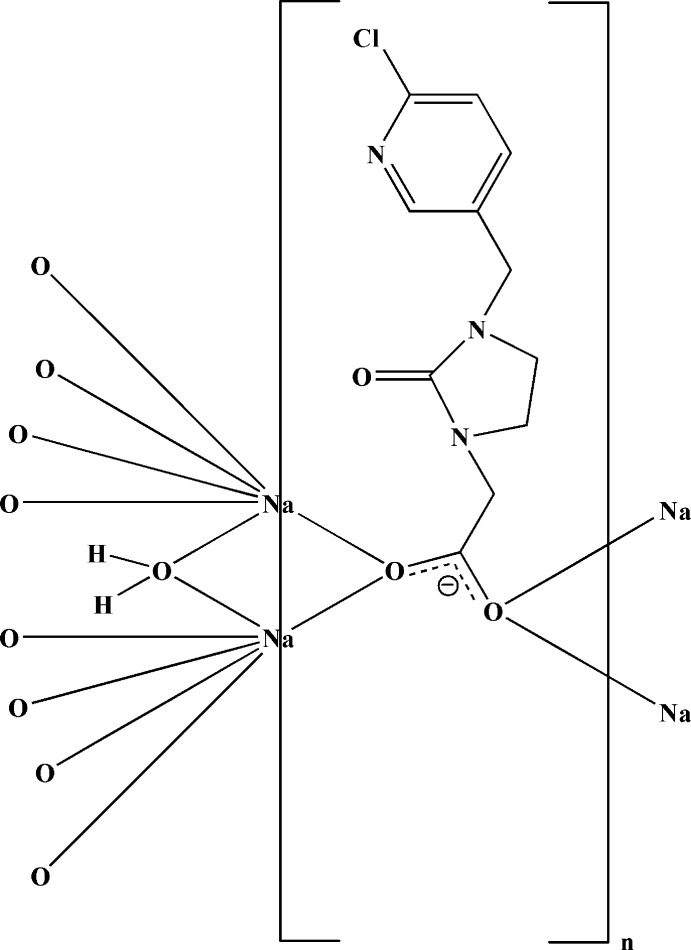


## Experimental

### 

#### Crystal data

[Na(C_11_H_11_ClN_3_O_3_)(H_2_O)]
*M* *_r_* = 309.68
Monoclinic, 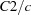

*a* = 45.655 (2) Å
*b* = 4.9113 (2) Å
*c* = 12.5205 (7) Å
β = 102.184 (5)°
*V* = 2744.2 (2) Å^3^
*Z* = 8
Mo *K*α radiation
μ = 0.33 mm^−1^
*T* = 293 K
0.3 × 0.2 × 0.1 mm

#### Data collection

Oxford Diffraction Xcalibur Sapphire3 diffractometer
Absorption correction: multi-scan (*CrysAlis PRO*; Oxford Diffraction, 2010 ▶) *T*_min_ = 0.836, *T*_max_ = 1.000
9498 measured reflections
2678 independent reflections
1909 reflections with *I* > 2σ(*I*)
*R*_int_ = 0.038

#### Refinement

*R*[*F* ^2^ > 2σ(*F* ^2^)] = 0.046
*wR*(*F* ^2^) = 0.112
*S* = 1.02
2678 reflections
191 parameters
H atoms treated by a mixture of independent and constrained refinement
Δρ_max_ = 0.44 e Å^−3^
Δρ_min_ = −0.46 e Å^−3^

### 

Data collection: *CrysAlis PRO* (Oxford Diffraction, 2010 ▶); cell refinement: *CrysAlis PRO*; data reduction: *CrysAlis PRO*; program(s) used to solve structure: *SHELXS97* (Sheldrick, 2008 ▶); program(s) used to refine structure: *SHELXL97* (Sheldrick, 2008 ▶); molecular graphics: *ORTEP-3* (Farrugia, 1997 ▶); software used to prepare material for publication: *PLATON* (Spek, 2009 ▶).

## Supplementary Material

Crystal structure: contains datablock(s) I, global. DOI: 10.1107/S1600536812025007/lh5481sup1.cif

Structure factors: contains datablock(s) I. DOI: 10.1107/S1600536812025007/lh5481Isup2.hkl

Additional supplementary materials:  crystallographic information; 3D view; checkCIF report

## Figures and Tables

**Table 1:** Hydrogen-bond geometry (Å, °)

*D*—H⋯*A*	*D*—H	H⋯*A*	*D*⋯*A*	*D*—H⋯*A*
O1*W*—H1*W*⋯O12^i^	0.80 (4)	2.02 (4)	2.826 (3)	179 (3)
O1*W*—H2*W*⋯O15^ii^	0.84 (4)	2.02 (4)	2.822 (2)	158 (3)
C10—H10*B*⋯O12^iii^	0.97	2.54	3.279 (3)	133
C13—H13*B*⋯O16^ii^	0.97	2.49	3.265 (3)	137

Symmetry codes: (i) 
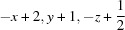
; (ii) 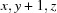; (iii) 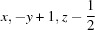.

**Fig. 1.**
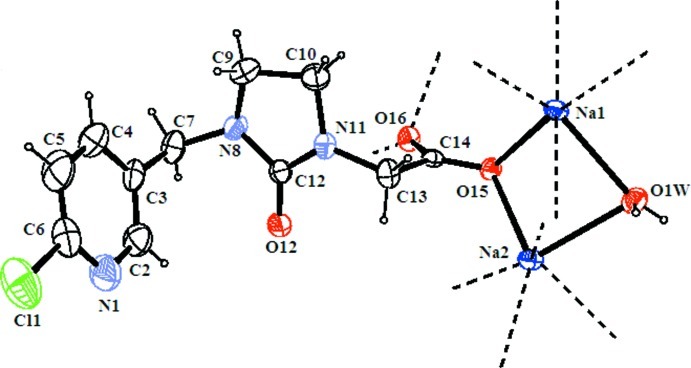
The asymmetric unit with ellipsoids are drawn at the 40% probability level. H atoms are shown as small spheres of arbitrary radii.

**Fig. 2.**
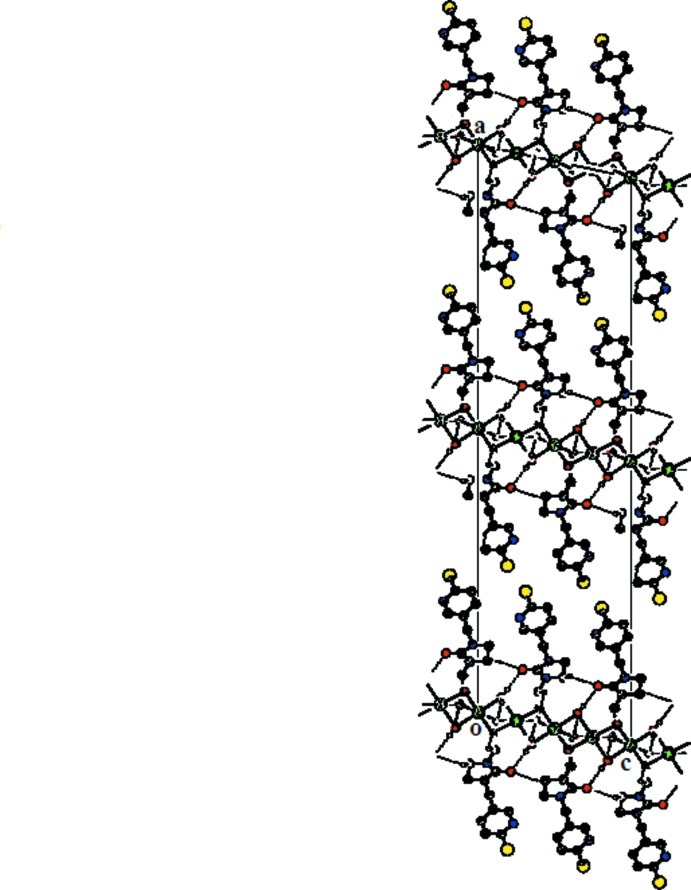
Part of the crystal structure. The broken lines show the intermolecular C—H···O and O—H···O interactions. Only H atoms involved in hydrogen bonds have been shown.

## supplementary crystallographic information

### Comment

For the development of nicotinoid insecticides the crucial turning-point could be traced back to the work done by the scientists from Nihon Tokushu Noyaku Seizo K and Nippon Bayer (Legocki & Polec, 2008). Insects become resistant to insecticides due to continuous use and hence it is imperative to introduce new molecules having novel mode of action (Kovganko & Kashkan, 2004). The outstanding development of neonicotinoid insecticides has been achieved for the modern crop protection, consumer products, and animal health markets between 1990 and today reflects the enormous importance of this chemical class (Zhao *et al.*, 2009). Neonicotinoids have low toxicity toward mammals and no teratogenic or mutagenic effects (Xu *et al.*, 2010). The biological activity and agricultural uses of neonicotinoid insecticides are enormous (Zhao *et al.*, 2009). From investigations it is revealed that the neonicotinoids are converted into numerous and variable metabolites in plants as well as in mammals (Tanner *et al.*, 2010).

The asymmetric unit is shown in Fig. 1. The bond lengths and angles observed in (I) show normal values and are comparable to those in related structures (Kapoor *et al.*, 2011; Kant *et al.*, 2012). There are two independent Na^I^ ions, one of which lies on an inversion center and is coordinated in a slightly disotorted octahedral environment. The other Na^I^ ion lies on a twofold rotation axis and is cooordinated in a slightly distorted trigonal bipyramidal coordination environment. In the organic ligand the imidazole ring adopts *half-chair* conformation (asymmetry parameter: ΔC_2_(C9—C10) = 2.31). The Na^I^ ions bridge organic ligands and solvent water molecules to form a two-dimensional structure parallel to (100). There are intermolecular O—H···O and weak C—H···O hydrogen bonds within the two-dimensional structure.

### Experimental

Ethyl[3-[(6-chloropyridin-3-yl)methyl]-2-(nitroimino)imidazolidin -1-yl]acetate (0.341 g m, 0.001 mol) was dissolved in 5 ml methanol and 5 ml 1 N NaOH solution was added. The reaction mixture was refluxed on a water bath at 343K for 12 h, and then cooled. The compound was re-precipitated upon neutralization with 1 N HCl. The compound was dissolved in methanol and crystallized in a fume hood at room temperature by the process of slow evaporation.

m.p. 575 K IR (KBr) ν_max_: 3421, 3300, 2872, 2930, 1668, 1606 cm^-1^. LC—MS/MS: 270, 252, 224, 149, 126 m/*z*.

### Refinement

All H atoms except water H atoms were positioned geometrically and were treated as riding on their parent C atoms, with C—H distances of 0.93–0.97 Å and with *U*_iso_(H) = 1.2*U*_eq_(C). Water H atoms were found in a difference map and isotropically refined

### Crystal data

**Table:**

[Na(C_11_H_11_ClN_3_O_3_)(H_2_O)]	*F*(000) = 1280
*M**_r_* = 309.68	*D*_x_ = 1.499 Mg m^−^^3^
Monoclinic, *C*2/*c*	Mo *K*α radiation, λ = 0.71073 Å
Hall symbol: -C 2yc	Cell parameters from 5286 reflections
*a* = 45.655 (2) Å	θ = 3.6–29.0°
*b* = 4.9113 (2) Å	µ = 0.33 mm^−^^1^
*c* = 12.5205 (7) Å	*T* = 293 K
β = 102.184 (5)°	Block, white
*V* = 2744.2 (2) Å^3^	0.3 × 0.2 × 0.1 mm
*Z* = 8	

### Data collection

**Table:**

Oxford Diffraction Xcalibur Sapphire3 diffractometer	2678 independent reflections
Radiation source: fine-focus sealed tube	1909 reflections with *I* > 2σ(*I*)
Graphite monochromator	*R*_int_ = 0.038
Detector resolution: 16.1049 pixels mm^-1^	θ_max_ = 26.0°, θ_min_ = 3.6°
ω scan	*h* = −56→52
Absorption correction: multi-scan (*CrysAlis PRO*; Oxford Diffraction, 2010)	*k* = −6→5
*T*_min_ = 0.836, *T*_max_ = 1.000	*l* = −15→15
9498 measured reflections	

### Refinement

**Table:**

Refinement on *F*^2^	Primary atom site location: structure-invariant direct methods
Least-squares matrix: full	Secondary atom site location: difference Fourier map
*R*[*F*^2^ > 2σ(*F*^2^)] = 0.046	Hydrogen site location: inferred from neighbouring sites
*wR*(*F*^2^) = 0.112	H atoms treated by a mixture of independent and constrained refinement
*S* = 1.02	*w* = 1/[σ^2^(*F*_o_^2^) + (0.0459*P*)^2^ + 2.523*P*] where *P* = (*F*_o_^2^ + 2*F*_c_^2^)/3
2678 reflections	(Δ/σ)_max_ = 0.001
191 parameters	Δρ_max_ = 0.44 e Å^−^^3^
0 restraints	Δρ_min_ = −0.46 e Å^−^^3^

### Special details

**Table:**

Experimental. *CrysAlis PRO*, Oxford Diffraction Ltd., Version 1.171.34.40 (release 27–08-2010 CrysAlis171. NET) (compiled Aug 27 2010,11:50:40) Empirical absorption correction using spherical harmonics, implemented in SCALE3 ABSPACK scaling algorithm.
Geometry. All e.s.d.'s (except the e.s.d. in the dihedral angle between two l.s. planes) are estimated using the full covariance matrix. The cell e.s.d.'s are taken into account individually in the estimation of e.s.d.'s in distances, angles and torsion angles; correlations between e.s.d.'s in cell parameters are only used when they are defined by crystal symmetry. An approximate (isotropic) treatment of cell e.s.d.'s is used for estimating e.s.d.'s involving l.s. planes.
Refinement. Refinement of *F*^2^ against ALL reflections. The weighted *R*-factor *wR* and goodness of fit *S* are based on *F*^2^, conventional *R*-factors *R* are based on *F*, with *F* set to zero for negative *F*^2^. The threshold expression of *F*^2^ > σ(*F*^2^) is used only for calculating *R*-factors(gt) *etc*. and is not relevant to the choice of reflections for refinement. *R*-factors based on *F*^2^ are statistically about twice as large as those based on *F*, and *R*- factors based on ALL data will be even larger.

### Fractional atomic coordinates and isotropic or equivalent isotropic displacement parameters (Å^2^)

**Table:**

	*x*	*y*	*z*	*U*_iso_*/*U*_eq_	
Na1	1.0000	1.0000	0.0000	0.0267 (3)	
Na2	1.0000	1.0028 (2)	0.2500	0.0286 (3)	
Cl1	0.76939 (2)	0.7478 (2)	0.18991 (10)	0.0963 (4)	
N1	0.81725 (5)	0.4509 (6)	0.2281 (2)	0.0652 (7)	
C2	0.81005 (7)	0.2440 (7)	0.0194 (3)	0.0685 (9)	
H2	0.8072	0.1756	−0.0513	0.082*	
C3	0.83467 (6)	0.1665 (5)	0.0972 (2)	0.0447 (7)	
C4	0.83683 (7)	0.2781 (6)	0.1992 (3)	0.0570 (8)	
H4	0.8534	0.2292	0.2529	0.068*	
C5	0.78956 (7)	0.4245 (8)	0.0471 (3)	0.0769 (11)	
H5	0.7728	0.4802	−0.0043	0.092*	
C6	0.79453 (7)	0.5174 (7)	0.1510 (3)	0.0614 (8)	
C7	0.85914 (6)	−0.0155 (5)	0.0735 (2)	0.0491 (7)	
H7A	0.8676	−0.1189	0.1387	0.059*	
H7B	0.8505	−0.1439	0.0167	0.059*	
N8	0.88292 (4)	0.1344 (4)	0.03879 (16)	0.0377 (5)	
C9	0.87821 (7)	0.2620 (6)	−0.0683 (2)	0.0549 (8)	
H9A	0.8602	0.3723	−0.0825	0.066*	
H9B	0.8770	0.1275	−0.1257	0.066*	
C10	0.90605 (6)	0.4362 (6)	−0.0581 (2)	0.0512 (7)	
H10A	0.9219	0.3394	−0.0829	0.061*	
H10B	0.9018	0.6046	−0.0989	0.061*	
N11	0.91385 (4)	0.4864 (4)	0.05842 (16)	0.0331 (5)	
C12	0.90251 (5)	0.2884 (5)	0.11303 (19)	0.0299 (5)	
O12	0.90778 (4)	0.2517 (4)	0.21182 (13)	0.0437 (5)	
C13	0.94038 (5)	0.6403 (5)	0.1062 (2)	0.0360 (6)	
H13A	0.9397	0.6836	0.1812	0.043*	
H13B	0.9401	0.8108	0.0668	0.043*	
C14	0.96972 (5)	0.4954 (4)	0.10520 (16)	0.0228 (5)	
O15	0.99295 (3)	0.6404 (3)	0.12325 (12)	0.0268 (4)	
O16	0.96893 (3)	0.2446 (3)	0.09003 (13)	0.0302 (4)	
O1W	1.03482 (4)	1.2101 (4)	0.15005 (15)	0.0364 (4)	
H1W	1.0512 (7)	1.220 (6)	0.189 (3)	0.055 (9)*	
H2W	1.0263 (8)	1.361 (8)	0.152 (3)	0.082 (12)*	

### Atomic displacement parameters (Å^2^)

**Table:**

	*U*^11^	*U*^22^	*U*^33^	*U*^12^	*U*^13^	*U*^23^
Na1	0.0343 (7)	0.0252 (6)	0.0226 (6)	−0.0001 (5)	0.0104 (5)	−0.0014 (5)
Na2	0.0387 (7)	0.0243 (6)	0.0236 (6)	0.000	0.0084 (5)	0.000
Cl1	0.0693 (6)	0.1034 (8)	0.1248 (9)	0.0315 (5)	0.0398 (6)	0.0245 (6)
N1	0.0462 (15)	0.0806 (19)	0.0682 (18)	0.0095 (14)	0.0111 (13)	0.0045 (15)
C2	0.0438 (18)	0.095 (3)	0.059 (2)	−0.0028 (18)	−0.0058 (15)	−0.0005 (19)
C3	0.0307 (13)	0.0456 (15)	0.0556 (18)	−0.0117 (12)	0.0042 (12)	0.0062 (13)
C4	0.0397 (16)	0.073 (2)	0.0546 (19)	0.0058 (15)	0.0004 (13)	0.0096 (16)
C5	0.0401 (18)	0.104 (3)	0.079 (3)	0.0158 (18)	−0.0045 (17)	0.019 (2)
C6	0.0423 (17)	0.067 (2)	0.078 (2)	0.0010 (15)	0.0184 (16)	0.0143 (18)
C7	0.0412 (15)	0.0387 (14)	0.0650 (19)	−0.0123 (13)	0.0060 (13)	−0.0015 (14)
N8	0.0363 (11)	0.0375 (11)	0.0369 (12)	−0.0057 (10)	0.0024 (9)	−0.0023 (9)
C9	0.0507 (17)	0.074 (2)	0.0349 (16)	−0.0085 (15)	−0.0032 (13)	−0.0032 (14)
C10	0.0439 (15)	0.074 (2)	0.0327 (15)	−0.0043 (14)	0.0015 (12)	0.0151 (14)
N11	0.0278 (10)	0.0322 (10)	0.0386 (11)	−0.0006 (9)	0.0057 (8)	0.0036 (9)
C12	0.0242 (11)	0.0327 (12)	0.0327 (14)	0.0049 (10)	0.0057 (10)	−0.0007 (10)
O12	0.0328 (9)	0.0672 (12)	0.0308 (10)	−0.0053 (8)	0.0057 (7)	0.0033 (9)
C13	0.0331 (13)	0.0251 (11)	0.0517 (16)	0.0000 (10)	0.0133 (11)	−0.0013 (11)
C14	0.0302 (11)	0.0213 (10)	0.0173 (10)	0.0011 (10)	0.0063 (9)	0.0016 (9)
O15	0.0288 (8)	0.0230 (7)	0.0283 (8)	−0.0029 (7)	0.0056 (6)	−0.0017 (6)
O16	0.0329 (9)	0.0182 (7)	0.0410 (9)	−0.0002 (6)	0.0116 (7)	−0.0023 (6)
O1W	0.0315 (10)	0.0405 (11)	0.0347 (10)	−0.0025 (9)	0.0011 (8)	−0.0037 (8)

### Geometric parameters (Å, º)

**Table:**

Na1—O16^i^	2.3239 (14)	C5—C6	1.351 (5)
Na1—O16^ii^	2.3239 (14)	C5—H5	0.9300
Na1—O15^iii^	2.4112 (14)	C7—N8	1.452 (3)
Na1—O15	2.4112 (14)	C7—H7A	0.9700
Na1—O1W^iii^	2.4208 (18)	C7—H7B	0.9700
Na1—O1W	2.4208 (18)	N8—C12	1.373 (3)
Na1—Na2^iii^	3.1302 (2)	N8—C9	1.454 (3)
Na1—Na2	3.1302 (2)	C9—C10	1.515 (4)
Na2—O15	2.3611 (16)	C9—H9A	0.9700
Na2—O15^iv^	2.3611 (16)	C9—H9B	0.9700
Na2—O1W	2.4432 (18)	C10—N11	1.448 (3)
Na2—O1W^iv^	2.4432 (18)	C10—H10A	0.9700
Na2—O16^v^	2.4969 (16)	C10—H10B	0.9700
Na2—O16^ii^	2.4968 (16)	N11—C12	1.353 (3)
Na2—Na1^iv^	3.1302 (2)	N11—C13	1.446 (3)
Na2—H2W	2.58 (3)	C12—O12	1.223 (3)
Cl1—C6	1.753 (3)	C13—C14	1.519 (3)
N1—C6	1.301 (4)	C13—H13A	0.9700
N1—C4	1.336 (4)	C13—H13B	0.9700
C2—C3	1.377 (4)	C14—O16	1.246 (2)
C2—C5	1.385 (5)	C14—O15	1.257 (2)
C2—H2	0.9300	O16—Na1^vi^	2.3239 (14)
C3—C4	1.374 (4)	O16—Na2^vi^	2.4968 (16)
C3—C7	1.509 (4)	O1W—H1W	0.81 (3)
C4—H4	0.9300	O1W—H2W	0.84 (4)
			
O16^i^—Na1—O16^ii^	180.00 (5)	C6—N1—C4	115.7 (3)
O16^i^—Na1—O15^iii^	83.72 (5)	C3—C2—C5	119.6 (3)
O16^ii^—Na1—O15^iii^	96.28 (5)	C3—C2—H2	120.2
O16^i^—Na1—O15	96.28 (5)	C5—C2—H2	120.2
O16^ii^—Na1—O15	83.72 (5)	C4—C3—C2	115.8 (3)
O15^iii^—Na1—O15	180.00 (7)	C4—C3—C7	120.6 (2)
O16^i^—Na1—O1W^iii^	76.78 (6)	C2—C3—C7	123.5 (3)
O16^ii^—Na1—O1W^iii^	103.22 (6)	N1—C4—C3	125.7 (3)
O15^iii^—Na1—O1W^iii^	88.29 (6)	N1—C4—H4	117.1
O15—Na1—O1W^iii^	91.71 (6)	C3—C4—H4	117.1
O16^i^—Na1—O1W	103.22 (6)	C6—C5—C2	118.2 (3)
O16^ii^—Na1—O1W	76.78 (6)	C6—C5—H5	120.9
O15^iii^—Na1—O1W	91.71 (6)	C2—C5—H5	120.9
O15—Na1—O1W	88.29 (6)	N1—C6—C5	125.1 (3)
O1W^iii^—Na1—O1W	180.00 (8)	N1—C6—Cl1	115.0 (3)
O16^i^—Na1—Na2^iii^	51.96 (4)	C5—C6—Cl1	119.9 (3)
O16^ii^—Na1—Na2^iii^	128.04 (4)	N8—C7—C3	112.9 (2)
O15^iii^—Na1—Na2^iii^	48.32 (4)	N8—C7—H7A	109.0
O15—Na1—Na2^iii^	131.68 (4)	C3—C7—H7A	109.0
O1W^iii^—Na1—Na2^iii^	50.26 (4)	N8—C7—H7B	109.0
O1W—Na1—Na2^iii^	129.74 (4)	C3—C7—H7B	109.0
O16^i^—Na1—Na2	128.04 (4)	H7A—C7—H7B	107.8
O16^ii^—Na1—Na2	51.96 (4)	C12—N8—C7	119.9 (2)
O15^iii^—Na1—Na2	131.68 (4)	C12—N8—C9	109.6 (2)
O15—Na1—Na2	48.32 (4)	C7—N8—C9	121.3 (2)
O1W^iii^—Na1—Na2	129.74 (4)	N8—C9—C10	102.0 (2)
O1W—Na1—Na2	50.26 (4)	N8—C9—H9A	111.4
Na2^iii^—Na1—Na2	180.00 (4)	C10—C9—H9A	111.4
O15—Na2—O15^iv^	82.15 (8)	N8—C9—H9B	111.4
O15—Na2—O1W	88.91 (5)	C10—C9—H9B	111.4
O15^iv^—Na2—O1W	130.33 (6)	H9A—C9—H9B	109.2
O15—Na2—O1W^iv^	130.33 (6)	N11—C10—C9	101.8 (2)
O15^iv^—Na2—O1W^iv^	88.91 (6)	N11—C10—H10A	111.4
O1W—Na2—O1W^iv^	130.75 (10)	C9—C10—H10A	111.4
O15—Na2—O16^v^	150.63 (6)	N11—C10—H10B	111.4
O15^iv^—Na2—O16^v^	81.11 (5)	C9—C10—H10B	111.4
O1W—Na2—O16^v^	83.79 (6)	H10A—C10—H10B	109.3
O1W^iv^—Na2—O16^v^	73.25 (6)	C12—N11—C13	122.9 (2)
O15—Na2—O16^ii^	81.11 (5)	C12—N11—C10	110.4 (2)
O15^iv^—Na2—O16^ii^	150.63 (6)	C13—N11—C10	120.80 (19)
O1W—Na2—O16^ii^	73.25 (6)	O12—C12—N11	127.1 (2)
O1W^iv^—Na2—O16^ii^	83.79 (6)	O12—C12—N8	124.4 (2)
O16^v^—Na2—O16^ii^	123.21 (8)	N11—C12—N8	108.5 (2)
O15—Na2—Na1^iv^	129.79 (5)	N11—C13—C14	114.54 (18)
O15^iv^—Na2—Na1^iv^	49.71 (3)	N11—C13—H13A	108.6
O1W—Na2—Na1^iv^	130.64 (5)	C14—C13—H13A	108.6
O1W^iv^—Na2—Na1^iv^	49.63 (4)	N11—C13—H13B	108.6
O16^v^—Na2—Na1^iv^	47.14 (3)	C14—C13—H13B	108.6
O16^ii^—Na2—Na1^iv^	133.19 (4)	H13A—C13—H13B	107.6
O15—Na2—Na1	49.71 (3)	O16—C14—O15	125.69 (19)
O15^iv^—Na2—Na1	129.79 (5)	O16—C14—C13	117.84 (18)
O1W—Na2—Na1	49.63 (4)	O15—C14—C13	116.43 (18)
O1W^iv^—Na2—Na1	130.65 (5)	C14—O15—Na2	122.53 (12)
O16^v^—Na2—Na1	133.19 (4)	C14—O15—Na1	121.41 (12)
O16^ii^—Na2—Na1	47.14 (3)	Na2—O15—Na1	81.97 (5)
Na1^iv^—Na2—Na1	179.49 (4)	C14—O16—Na1^vi^	125.73 (13)
O15—Na2—H2W	101.9 (8)	C14—O16—Na2^vi^	110.77 (13)
O15^iv^—Na2—H2W	145.3 (8)	Na1^vi^—O16—Na2^vi^	80.89 (5)
O1W—Na2—H2W	19.0 (8)	Na1—O1W—Na2	80.11 (5)
O1W^iv^—Na2—H2W	112.1 (8)	Na1—O1W—H1W	151 (2)
O16^v^—Na2—H2W	79.3 (8)	Na2—O1W—H1W	110 (2)
O16^ii^—Na2—H2W	62.4 (8)	Na1—O1W—H2W	100 (2)
Na1^iv^—Na2—H2W	125.2 (8)	Na2—O1W—H2W	90 (2)
Na1—Na2—H2W	55.2 (8)	H1W—O1W—H2W	108 (3)
			
O16^i^—Na1—Na2—O15	−59.31 (6)	C13—N11—C12—N8	−163.81 (19)
O16^ii^—Na1—Na2—O15	120.69 (6)	C10—N11—C12—N8	−10.9 (3)
O15^iii^—Na1—Na2—O15	180.000 (3)	C7—N8—C12—O12	23.0 (3)
O1W^iii^—Na1—Na2—O15	46.52 (8)	C9—N8—C12—O12	170.2 (2)
O1W—Na1—Na2—O15	−133.48 (8)	C7—N8—C12—N11	−155.4 (2)
O16^i^—Na1—Na2—O15^iv^	−39.26 (7)	C9—N8—C12—N11	−8.1 (3)
O16^ii^—Na1—Na2—O15^iv^	140.74 (7)	C12—N11—C13—C14	80.5 (3)
O15^iii^—Na1—Na2—O15^iv^	−159.94 (9)	C10—N11—C13—C14	−69.7 (3)
O15—Na1—Na2—O15^iv^	20.06 (9)	N11—C13—C14—O16	−17.1 (3)
O1W^iii^—Na1—Na2—O15^iv^	66.58 (8)	N11—C13—C14—O15	164.93 (18)
O1W—Na1—Na2—O15^iv^	−113.42 (8)	O16—C14—O15—Na2	−140.66 (17)
O16^i^—Na1—Na2—O1W	74.17 (8)	C13—C14—O15—Na2	37.1 (2)
O16^ii^—Na1—Na2—O1W	−105.83 (8)	O16—C14—O15—Na1	118.07 (19)
O15^iii^—Na1—Na2—O1W	−46.52 (8)	C13—C14—O15—Na1	−64.2 (2)
O15—Na1—Na2—O1W	133.48 (8)	O15^iv^—Na2—O15—C14	73.13 (14)
O1W^iii^—Na1—Na2—O1W	180.000 (2)	O1W—Na2—O15—C14	−155.86 (15)
O16^i^—Na1—Na2—O1W^iv^	−172.30 (8)	O1W^iv^—Na2—O15—C14	−8.69 (18)
O16^ii^—Na1—Na2—O1W^iv^	7.70 (8)	O16^v^—Na2—O15—C14	128.87 (15)
O15^iii^—Na1—Na2—O1W^iv^	67.01 (9)	O16^ii^—Na2—O15—C14	−82.65 (15)
O15—Na1—Na2—O1W^iv^	−112.99 (9)	Na1^iv^—Na2—O15—C14	57.82 (16)
O1W^iii^—Na1—Na2—O1W^iv^	−66.47 (14)	Na1—Na2—O15—C14	−122.29 (16)
O1W—Na1—Na2—O1W^iv^	113.53 (14)	O15^iv^—Na2—O15—Na1	−164.57 (7)
O16^i^—Na1—Na2—O16^v^	81.15 (10)	O1W—Na2—O15—Na1	−33.57 (6)
O16^ii^—Na1—Na2—O16^v^	−98.85 (10)	O1W^iv^—Na2—O15—Na1	113.60 (7)
O15^iii^—Na1—Na2—O16^v^	−39.54 (8)	O16^v^—Na2—O15—Na1	−108.84 (10)
O15—Na1—Na2—O16^v^	140.46 (8)	O16^ii^—Na2—O15—Na1	39.65 (4)
O1W^iii^—Na1—Na2—O16^v^	−173.02 (8)	Na1^iv^—Na2—O15—Na1	−179.885 (9)
O1W—Na1—Na2—O16^v^	6.98 (8)	O16^i^—Na1—O15—C14	−99.57 (14)
O16^i^—Na1—Na2—O16^ii^	180.000 (1)	O16^ii^—Na1—O15—C14	80.43 (14)
O15^iii^—Na1—Na2—O16^ii^	59.31 (6)	O1W^iii^—Na1—O15—C14	−22.69 (15)
O15—Na1—Na2—O16^ii^	−120.69 (6)	O1W—Na1—O15—C14	157.31 (15)
O1W^iii^—Na1—Na2—O16^ii^	−74.17 (8)	Na2^iii^—Na1—O15—C14	−56.62 (15)
O1W—Na1—Na2—O16^ii^	105.83 (8)	Na2—Na1—O15—C14	123.38 (15)
C5—C2—C3—C4	0.2 (4)	O16^i^—Na1—O15—Na2	137.04 (5)
C5—C2—C3—C7	−176.3 (3)	O16^ii^—Na1—O15—Na2	−42.96 (5)
C6—N1—C4—C3	−0.6 (5)	O1W^iii^—Na1—O15—Na2	−146.07 (6)
C2—C3—C4—N1	0.3 (5)	O1W—Na1—O15—Na2	33.93 (6)
C7—C3—C4—N1	176.9 (3)	Na2^iii^—Na1—O15—Na2	180.000 (2)
C3—C2—C5—C6	−0.4 (5)	O15—C14—O16—Na1^vi^	−35.2 (3)
C4—N1—C6—C5	0.4 (5)	C13—C14—O16—Na1^vi^	147.11 (16)
C4—N1—C6—Cl1	−179.1 (2)	O15—C14—O16—Na2^vi^	58.5 (2)
C2—C5—C6—N1	0.1 (6)	C13—C14—O16—Na2^vi^	−119.20 (17)
C2—C5—C6—Cl1	179.6 (3)	O16^i^—Na1—O1W—Na2	−128.89 (6)
C4—C3—C7—N8	−87.5 (3)	O16^ii^—Na1—O1W—Na2	51.11 (6)
C2—C3—C7—N8	88.8 (3)	O15^iii^—Na1—O1W—Na2	147.16 (6)
C3—C7—N8—C12	71.7 (3)	O15—Na1—O1W—Na2	−32.83 (6)
C3—C7—N8—C9	−71.6 (3)	Na2^iii^—Na1—O1W—Na2	180.000 (1)
C12—N8—C9—C10	22.3 (3)	O15—Na2—O1W—Na1	33.61 (5)
C7—N8—C9—C10	169.0 (2)	O15^iv^—Na2—O1W—Na1	112.35 (8)
N8—C9—C10—N11	−26.8 (3)	O1W^iv^—Na2—O1W—Na1	−113.33 (6)
C9—C10—N11—C12	24.0 (3)	O16^v^—Na2—O1W—Na1	−174.89 (6)
C9—C10—N11—C13	177.6 (2)	O16^ii^—Na2—O1W—Na1	−47.43 (5)
C13—N11—C12—O12	17.9 (3)	Na1^iv^—Na2—O1W—Na1	179.44 (4)
C10—N11—C12—O12	170.9 (2)		

Symmetry codes: (i) −*x*+2, −*y*+1, −*z*; (ii) *x*, *y*+1, *z*; (iii) −*x*+2, −*y*+2, −*z*; (iv) −*x*+2, *y*, −*z*+1/2; (v) −*x*+2, *y*+1, −*z*+1/2; (vi) *x*, *y*−1, *z*.

### Hydrogen-bond geometry (Å, º)

**Table:**

*D*—H···*A*	*D*—H	H···*A*	*D*···*A*	*D*—H···*A*
O1*W*—H1*W*···O12^v^	0.80 (4)	2.02 (4)	2.826 (3)	179 (3)
O1*W*—H2*W*···O15^ii^	0.84 (4)	2.02 (4)	2.822 (2)	158 (3)
C10—H10*B*···O12^vii^	0.97	2.54	3.279 (3)	133
C13—H13*B*···O16^ii^	0.97	2.49	3.265 (3)	137

Symmetry codes: (ii) *x*, *y*+1, *z*; (v) −*x*+2, *y*+1, −*z*+1/2; (vii) *x*, −*y*+1, *z*−1/2.
